# The IRB structure and medical research reform

**DOI:** 10.1186/s40169-018-0188-3

**Published:** 2018-04-02

**Authors:** Julie Babyar

**Affiliations:** 1239 Symphony Way, Vallejo, CA 94191 USA

## Abstract

Optimal Independent Review Board (IRB) structure encompasses ongoing process improvement, ethics policies and continuous relationship building, all sound in evidence. With optimal IRB structure, a global research infrastructure will flourish. Evidence for IRB structure must be detailed and expert operational recommendations should guide. Too, health service research oversight should assist in funding as well as collaboration. A national and international research agenda will only benefit from best operations, guided in evidence, supported in best regulatory and research leadership practice. It is imperative that the IRB structure be reformed.

## Commentary

As global communities grow, healthcare shifts and organizations realize collaborative agreements in medicine, research in medicine will continue to be paramount. This critical component to healthcare must also grow in strength and quality. The structure and implementation of sound research protections and policies, which is currently the responsibility of Independent Review Boards (IRBs), must be reformed and improved upon. Specifically, the review process is an area for global unity in ethics and philosophy, and it is an opportunity to build stakeholder relationships in operational implementation.

Historically, ethical decisions within medical research have been suboptimal. Equally of concern, researchers have been less than forthcoming in operations, and researchers have been considered as foreigners to those they are studying. This lack of transparency in medicine and lack of oversight from governments contributed to poor trust among public and medical researchers.

In fact, international ethical guidelines were set in 1947 as a consequence of unethical experimentation on humans and prisoners of war. The Nuremberg Code was followed by the United Nations Declaration of Human Rights, and both of these sought to protect individual rights [1]. Because lack of transparency and poor ethical decisions continued to be made in medical research, declarations and policies worldwide expanded. The most prevailing oversight aligns with the Helsinki declaration, first adopted in 1964 by the World Medical Association and recently revised in 2013 [2].

Some countries provide stronger oversight than others, and the United States has a specific government branch for medical research protections (the Office for Human Research Protections) along with established federal regulations. Agencies such as the World Health Organization also offer strong guidance, resolutions and policy templates in effort to assure individual rights in medical research. Future collaborations in medical research provide an opportunity to enforce agreements and resolutions through sound and unbiased regulatory audit. Additionally, concerns that arise from new findings can provide process improvement opportunities worldwide.

The primary means to public assurance in medical research is through IRBs. IRBs review research proposals for regulation adherence and protection of individual welfare and rights. They also determine that all aspects of the research are ethical. IRBs consist of a minimum of 5 persons from various backgrounds, one member must be a community layperson and IRBs must register with the federal government. IRBs are independent and some are private. The process and workload for IRBs have grown over the years due to an increase in regulations and assurances they must oversee [3]. Additionally, IRB boards are inconsistent. They vary in thoroughness and cost of reviews. They also vary in quality of decision determination and time required for approval [4].

IRBs are grounded in policies, and policies structured to protect in human research are often accompanied by regulations that cast wide nets. Therefore, research implementation can be slower and efficiencies are less than ideal. Additionally, guidance provided from IRBs to researchers has been cited for lack of ample evidence, and this guidance is not often accompanied by enforcement or consequences to violations. Finally, neither review approvals nor agreements between healthcare and governments will provide the actual work needed for sustained community and stakeholder relations. Process improvement strategies can work toward addressing these issues.

The primary strategy to improve research protections and review lies within the foundation of trust. While numerous studies on trust and medicine are inconsistent and often sample small populations in the United States, general undertones of distrust in medicine prevail. This is unfortunate for the global public, disappointing for the health professions and frustrating for governing bodies. Trust and accompanying relationships, while encouraged in clinicians at the bedside, are equally critical within the medical research field. The IRB process, the initial stage of medical research review and community impact assessment, is the first impression toward all stakeholders involved. Researchers must be forthcoming to investors, community leaders and stakeholders on data, methods and value of the research. Healthcare providers and patients must communicate concerns, anticipate needs and address cultural differences before establishing a research foundation. Removal of familiarity, bias and assumption must be at the forefront of IRB and academic medicine affiliations. Industry and academia must also have faith in expertise and knowledge of the reviewers, without conflict of interest. Ongoing dialogue, cultivated in consistent and compassionate approach, must be maintained by all invested parties throughout the review and approval process for medical research.

Ongoing changes in the healthcare system provide opportunity for efficiency and modification of the IRB process and design. The Clinical Trials Transformation Initiative recommends the use of one IRB for research that involves multiple sites, even though many researchers continue to use an IRB for every site [5]. Training in regulatory and ethical guidelines for IRB members has been suggested to increase efficiency [6]. Electronic IRB administrative systems assist researchers in streamlining and minimizing tasks [7].

Additionally, it has been documented that there are no published articles on the effectiveness of the IRB structure, and no evidence on process or outcomes related to IRB structure and research [8]. In fact, recent analyses found various IRB boards often asked for different and competing revisions when presented with the exact same studies. It has also been noted that some IRB decisions are not in line with federal policies and IRB approval wait times delay research. These same analyses found no evidence to support current or varying IRB structure [9]. The opportunity to require ongoing and definitive national research and data collection on IRBs should be immediately seized. Specifically, health service research should be completed on IRB structure, research approvals, and the outcomes, impact and effectiveness of these decisions. Evidence for IRB structure, or lack thereof, should be prioritized by health service research agencies and academia. Stakeholders and expert oversight can drive quality data as well as initiate and encourage structural changes recommended for IRBs.

As healthcare changes and grows, research demands and agenda will follow suit. Aspects of medical research protections, approvals and review must reform alongside these changes. Evidence for IRB structure must be detailed, operational recommendations provided by experts such as CTTI must be heeded and health service research oversight should assist in funding as well as collaboration. A national and international research agenda will only benefit from best operations, guided in evidence, at the regulatory and approval levels. Optimal IRB structure encompasses ongoing process improvement, ethics policies and continuous relationship building, all sound in evidence. With optimal IRB structure, a global research infrastructure will flourish.
